# Supplementary material for: Second-Generation Lignocellulosic Supportive Material Improves Atomic Ratios of C:O and H:O and Thermomechanical Behavior of Hybrid Non-Woody Pellets
Source: Molecules. 2020 Sep 15;25(18):4219. doi: 10.3390/molecules25184219 (PMC7570733; doi:10.3390/molecules25184219)
Supplement: Supplementary file 1 [file molecules-25-04219-s001.pdf]

Table S1. Descriptive analysis for the relative potential of the residual biomass as an alternative non-woody biomass waste to develop fuel pellets for heating and power.

Feedstock	Property									
	Proximal, %				Elemental, %					Physicothermal, MJ kg^{-1}
	Water	V_M	F_C	Ash	C	H	O	N	S	HHV
Residual biomass	12.2	69.8	24.3	5.85	53.0	6.0	39.5	0.0	1.3	21.8
Woody										
Pine sawdust [a]	7.4	86.3	13.5	0.2	45.2	6.3	48.2	0.1	0.0	20.0
Chestnut sawdust [a]	9.2	82.1	17.5	0.4	45.5	5.7	48.2	0.2	0.0	19.1
Eucalyptus sawdust [a]	10.5	84.6	14.9	0.5	46.8	6.1	46.5	0.1	0.0	19.5
Pine cone leaves [c]	10.1	76.5	22.4	1.1	52.9	6.1	39.5	0.4	0.0	20.9
Pine kernel shells [c]	9.4	78.4	19.8	1.8	52.3	6.2	38.9	0.6	0.0	20.8
Spruce [b]	-	-	-	0.5	47.1	6.0	46.8	0.0	0.0	19.5
Mean	7.0	61.6	12.8	0.7	48.5	6.1	44.7	0.2	0.0	20.0
Standard deviation	4.4	38.2	8.4	0.5	3.1	0.2	3.9	0.2	0.0	0.7
Non-woody										
Cellulosic residue [a]	4.4	87.7	11.0	1.3	41.0	6.4	51.0	0.3	0.0	17.6
Coffee husks [a]	6.7	79.4	16.1	4.5	43.2	6.3	43.2	2.6	0.2	20.1
Grape waste [a]	6.4	67.9	24.6	7.5	50.0	6.0	34.4	2.0	0.1	22.1
Reed canary grass [b]	-	-	-	2.2	45.4	5.9	48.4	0.2	0.0	19.1
Timothy hay [b]	-	-	-	3.8	45.5	5.9	48.4	0.2	0.0	18.2
Switchgrass [b]	-	-	-	3.6	45.0	6.0	48.8	0.1	0.0	18.5
Almond shells [c]	6.5	78.9	19.6	1.4	49.4	5.8	42.9	0.3	0.0	19.6
Coffee dregs [c]	7.2	70.2	21.2	8.6	47.9	5.6	33.9	3.6	0.3	19.4
Coffee husks [c]	8.4	79.2	16.7	4.2	49.5	6.1	36.9	2.9	0.2	20.2
Cocoa shells [c]	6.7	70.4	21.7	7.9	47.9	5.9	35.3	2.7	0.2	19.1
Grape pomace [c]	11.6	67.6	19.7	12.7	45.5	5.0	34.7	1.8	0.1	18.7
Hazelnut shells [c]	13.6	75.8	23.2	0.9	52.5	5.7	40.5	0.5	0.1	18.7
Miscanthus [c]	5.6	77.6	13.4	9.0	47.9	5.8	36.7	0.4	0.1	18.7
Olive stones [c]	4.3	81.5	17.9	0.6	51.2	6.0	41.9	0.3	0.0	20.5
Switchgrass [c]	12.4	79.7	15.7	4.6	47.8	5.7	41.0	0.8	0.1	18.9
Mean	8.7	75.3	18.7	6.1	48.8	5.7	37.6	1.6	0.1	19.3
Standard deviation	3.4	5.2	3.3	4.2	2.2	0.3	3.1	1.3	0.1	0.7
Fossil derivatives										
High-volatile bituminous coal [a]	1.4	37.7	54.7	7.6	77.9	5.1	6.2	1.7	1.5	32.4
Semianthracite [a]	0.8	7.5	67.0	25.5	66.8	1.1	3.6	1.1	0.5	25.6
Mean	1.1	22.6	60.9	16.6	72.4	3.1	4.9	1.4	1.0	29.0
Standard deviation	0.4	21.4	8.7	12.7	7.8	2.8	1.8	0.4	0.7	4.8
Average	7.8	72.6	22.2	4.5	49.8	5.7	39.4	0.9	0.2	20.4
Standard deviation	3.4	18.3	13.8	5.5	7.5	1.0	11.4	1.1	0.4	2.9

Volatile mater, V_M; fixed-carbon, F_C; higher heating value, HHV;

[a] Gil et al. [14];

[b] Harun and Afzal [13];

[c] Garcia et al. [7].

Table S2. Technical specifications of integrated set for pelleting.

Characteristic	Unit
Load capacity of feeder silo	250 kg
Engine potency	20 HP
Production capacity	250 kg h^{-1}
Nominal diameter of tungsten channel-forming die	200 mm
Nominal diameter of tungsten pressing rollers	2 x 100 mm
Maximum nominal temperature of die	150 °C
Diameter of pellet	6–8 mm
Apparent density of pellet	1000–1400 kg m^{-3}
Weight	850 kg
Dimensions of pelletizer machine	4200 mm x 2450 mm x 1750 mm

Table S3. Norms for the characterization of the starting materials.

Norm/ Method	Property	Instrument
ASTM E871-82	Water	Horizontal drying-oven (Marconi MAO35/5)
ASTM E871-82	Volatile material and fixed-carbon	Digital muffle furnace (SPlabor 1200DM/B)
ASTM D1102-84	Ash	Muffle furnace
NDF–ADF–ADL	Cellulose, hemicellulose, lignin, and extractive	Horizonal drying-oven; muffle furnace
EN 15104	C, H, O, and N	Elemental analyzer (Flash Smart CHNS/O)
EN 15289	S	Elemental analyzer
ASTM D5865-13	Higher heating value	Isothermal digital calorimeter (IKA C200)

American Society for Testing and Materials (ASTM);

European Norm (EN);

Neutral detergent fiber, acid detergent fiber, acid detergent liquid (NDF–ADF–ADL).
